# Effects of radiofrequency radiation in the presence of gold nanoparticles for the treatment of renal cell carcinoma

**DOI:** 10.15171/jrip.2017.20

**Published:** 2016-11-06

**Authors:** Safoora Nikzad, Golshan Mahmoudi, Payam Amini, Milad Baradaran-Ghahfarokhi, Akbar Vahdat-Moaddab, Seyedeh Maryam Sharafi, Leila Hojaji-Najafabadi, Ali Hosseinzadeh

**Affiliations:** ^1^Department of Medical Physics, Faculty of Medicine, Hamadan University of Medical Sciences, Hamadan, Iran; ^2^Medical Physics Department, School of Medicine, Sabzevar University of Medical Sciences, Sabzevar, Iran; ^3^Department of Epidemiology and Reproductive Health, Reproductive Epidemiology Research Center, Royan Institute for Reproductive Biomedicine, ACECR, Tehran, Iran; ^4^Department of Medical Physics and Medical Engineering & Students Research Committee, School of Medicine, Isfahan University of Medical Sciences, Isfahan, Iran; ^5^Department of Medical Radiation Engineering, Faculty of Advanced Sciences & Technologies, Isfahan University, Isfahan, Iran; ^6^Department of Radiology, Isfahan University of Medical Sciences, Isfahan, Iran; ^7^Infectious Disease and Tropical Medicine Research Center, Isfahan University of Medical Sciences, Isfahan, Iran; ^8^Nanotechnology Department, Faculty of Advanced Sciences and Technologies, Isfahan University, Isfahan, Iran; ^9^Research Center for Modeling in Health, Institute for Future Studies in Health, Kerman University of Medical Sciences, Kerman, and, Epidemiology and Biostatistics Department, School of Health, Sabzevar University of Medical Sciences, Sabzevar, Iran

**Keywords:** Synergic effects, Radiofrequency radiation, Gold nanoparticles, Renal cell carcinoma, Cancer

## Abstract

**Introduction:** The most common type of kidney cancer is renal cell carcinoma (RCC), which accounts for more than 80% of all kidney cancers.

**Objectives:** The aim of this study was to evaluate the effects of radiofrequency (RF) radiation in the presence of gold nanoparticles (GNPs) for the treatment of RCC.

**Materials and Methods:** Human embryonic kidney (HEK) cancer cells were divided into 6 groups. Various tests were performed on HEK cells in the presence of RF and GNPs. In order to investigate the radiation effects on the cells’ survival, MTT [3-(4,5-dimethylthiazol–2-yl)-2,5-iphenyltetrazolium bromide] assay was performed at different days during and post-irradiation period. The repeated measure analysis of variance (ANOVA) method was used for statistical analysis of the cells’ survival using SPSS version 16.0. A significant level of 0.05 was considered to the tests.

**Results:** Using the ANOVA test, a significant decrease in cell’s survival was seen in the RF exposed group 3 compared to the control group (*P*=0.035). While, differences were not significant between RF exposed group 2 and the control group (*P*>0.05). A significant decrease in cell’s survival in the RF exposed groups 5 (*P*=0.025) and 6 (*P*=0.018) at the presence of GNP compared to the control group was seen.

**Conclusion:** Results of this study showed that, this method can be efficiently used for RCC treatment as an alternative to nephrectomy. More follow up in vivo studies on mammalians are needed to investigate the potential of the presented method for clinical applications.

**Implication for health policy/practice/research/medical education:**

One of the most common types of kidney cancer is renal cell carcinoma (RCC), which accounts for more than 80% of all kidney cancers. Considering the difficulties associated with RCC treatment, new approaches are needed to enhance the radiotherapeutic efficiency. In this study the effect of radiofrequency (RF) radiation in the presence of gold nanoparticles (GNPs), as a novel treatment method for RCC treatment was evaluated. Results showed that, this method can be efficiently used for RCC treatment as an alternative of nephrectomy.

## Introduction

The most common type of kidney cancer is renal cell carcinoma (RCC), which accounts for more than 80% of all kidney cancers (1). RCC is a relatively rare tumor accounting for approximately 3% of malignancies in adults (1). Studies on RCC treatment have been shown that radical nephrectomy can be used as the gold standard for RCC treatment (1-3). Recent publications indicated that, for patients with localized RCC, partial nephrectomy in carefully selected patients may be an option (1-3). However, the probability of cure is related to the cancer stage and many patients deemed unsuitable for this kind of treatment due to their underlying preoperative conditions such as renal insufficiency, solitary kidney, and high-risk comorbidities (4-8). Therefore, new approaches are needed to enhance the therapeutic efficiency of RCC.

The use of radiofrequency (RF) radiation for medical applications plays an important role in human daily life (9-11). The effects of such radiation on human body depend on its frequency and power (9-11). In this regard, considering the biological effects, there has been an increasing interest on medical applications of RF radiation (12). RF radiation-induced free radical formation in biological tissues and cell lines has been reported is some literatures (9-11). According to recent reports, biological systems may interact resonantly with electromagnetic RF radiation. For such biological interactions, the reactive oxygen species (ROS) are indicated as constituent (14). Superoxide anion (O_2_), created by the mitochondria, is the main ROS that produces hydrogen peroxide (H_2_O_2_) by the action of superoxide dismutase (SOD) (14). It should be noted that catalase and glutathione peroxidase (GSH-Px) act as scavengers of ROD (15).

Therefore, RF radiation at significant energy and frequency can be used for treatment of RCC, since, malondialdehyde (MDA) is the breakdown product of the major chain reactions which result to polyunsaturated fatty acids oxidation and thus serves as a reliable marker of renal tissue oxidative stress-mediated lipid peroxidation (LPO) (16). Meanwhile, levels of these endogenous indices of oxidative stress in exposed animals have not yet been reported.

On the other hand, with the production of gold nanoparticles (GNPs) and the progress of nanotechnology that are of considerable biological compatibility, new horizons in cancer treatment have been opened up and GNPs have been proven effective in the treatment of disease (17). GNPs have both strong radiation absorption and higher stability (18-20). The high extinction coefficient of radiation by the GNPs which is due to the coherent oscillations of electrons in gold metal is intensified by inducing RF radiation (18-20). This phenomenon can be used in various applications such as hyperthermia of RCC (21). It should be noted that, for healthy tissues, the toxicity of GNPs can be determined by size, shape, surface charge, and surface coating, however, the overall toxicity dose of GNPs is in an acceptable level (17).

It has been reported that thermotherapy using microwave radiation in synergism with GNPs may be proposed as a new approach to treat leishmaniasis in future studies (21). Moreover, magnetic nanoparticles (MNPs) have been employed as a potent device in numerous biological and medical investigations (22). Based on some studies, the intracellular transportation of iron oxide nanoparticles has been proven to affect cell function (22,23). In other studies, GNPs have been utilized as catalysts, nonlinear optical devices and light storing tools (22,23).

This study aimed to evaluate the effects of RF radiation in the presence of GNPs for the treatment of RCC. To the best of our knowledge, no published articles have presented a study on this issue with the methodology and analysis described here.

## Materials and Methods

### Synthesis of gold nanoparticles

GNPs were synthesized according to standard wet chemical methods using sodium borohydride (24). Fifty milliliters of an aqueous solution containing 4.3 mg of solid sodium borohydride was added to 100 mL of tetrachloroauric acid with aqueous solution of 100-μmol/L. The solution was kept under vigorous stirring overnight (24). Then, the GNPs were filtered through 0.22 μm paper filter. The size of nanoparticle was investigated and calculated by the use of electron microscopy.

### Cell culture

Experiments were carried out on human embryonic kidney (HEK) cancer cells provided from Iran cell bank of Pasteur institute (Tehran, Iran). The cells were cultured in 25 mL culture flasks of Dulbecco’s Modified Eagle’s Medium (DMEM Gibco Laboratories, Cergy Pontoise, France) supplemented with 10% fetal bovine serum (FBS Gibco Laboratories, Cergy Pontoise, France), 2 mM glutamine, 100 U penicillin per mL, and 100 mg streptomycin per mL (Gibco Laboratories, Cergy Pontoise, France). Cells were grown in humidified cell incubator at 37°C under 5% CO_2_ atmosphere and 95% air.

### Experimental design

To perform the experiment, 96 well plates were used. Cells were seeded and the density of 5000 cell/well was put in plates. They were allowed to adhere and grown overnight in 200 μL medium.

At first, to determine the optimum nanoparticle concentration the cells were incubated with fresh medium containing serial concentrations (0 to 80 μM) of GNPs for 2 hours and a control group without treatment (25). The survival of the cells was investigated after 24 hours.

The cells were divided into 6 groups. For the first group (group 1), as control one, no radiation and GNP was applied. For the second and third groups (group 2 and 3), the cells were exposed to a RF simulator for 1 and 2 hour/day, respectively, for 8 days. For these two groups (group 2 and 3) GNP was not used. Moreover, to investigate the effects of GNPs on the cell death, three other groups were designed. For group 4, no radiation was applied and the cells were incubated with GNP for two hours. Groups 5 and 6 were exposed to the RF simulator for 1 and 2 hour/day, respectively, for 8 days. Both groups 5 and 6 were incubated with GNPs during the exposure time. Table 1 gives the studied groups and design of the experiment. The RF simulator (designed and produced at the Medical Physics Department, School of Medicine, Isfahan University of Medical Sciences) was adjusted on 1.0 W and 900 MHz frequency for the exposure. The distance between the simulator antenna and the wells was kept at 2.5 cm.

**Table 1:** The studied groups and design of the experiment

**Group**	**Irradiation**		**Gold nanoparticles**
	**1 hour per day**	**2 hour per day**	
1	-	-	-
2	√	-	-
3	-	√	-
4	-	-	√
5	√	-	√
6	-	√	√

In order to investigate the radiation effects on the cells’ survival, MTT [3-(4,5-dimethylthiazol–2-yl)-2,5-iphenyltetrazolium bromide] assay was performed at different days during and post-irradiation period .

### MTT solution

The cell’s viability was assessed using the MTT method. The MTT assay involves the conversion of the water-soluble MTT to an insoluble formazan. The MTTs was dissolved in sterile phosphate-buffered saline (PBS) at 5 mg/mL. Next, the filtration was performed using a 0.22 µm filter and then the solution was stored in dark condition at 4°C for a period lasting less than 3 weeks.

Five wells of a 96-well plate were used for every experimental condition. The medium was renewed every three days to avoid possible medium product inaccuracy. Since the cell lines were adhesive, their media could simply be renewed without making any damage to the cells. To perform the MTT assay, the MTT solution at appropriate concentrations (10 µL MTT solutions in each 100 µL media) was added to each well and the plates were then incubated at 37°C for 4 hours. The incubation was followed by formation of purple formazan salts crystals, from metabolically active cells. After the incubation, the remaining solution of the MTT was removed and 100 λ of dimethyl sulphoxide (DMSO) was added to each of the wells. This process was done in order to dissolve the formazan crystals. Next, the plates were shaken for 5 minutes on a plate shaker to ensure adequate solubility. Reading the absorbance of each well was done at 570 nm (single wavelength) using an ELISA reader (Stat Fax 2100, Awareness Technology, Inc., USA). The number of viable cells was directly correlated to the amount of purple formazan crystals formed. To avoid the variability inherent to the assay used, all tests were performed for three independent trials.

### Ethical issues

The research followed the tenets of the Declaration of Hel­sinki, and the study was approved by the ethical committee of Sabzevar University of Medical Sciences.

### Statistical analysis

Mean values and standard deviations were calculated and statistical significance of the differences between exposed and control groups were evaluated. Because the data were collected at various times and groups, the repeated measure analysis of variance (ANOVA) method was used. Statistical analysis was performed using SPSS version 16.0. A significant level of 0.05 was considered to the tests.

## Results

A size histogram curve of the GNPs, by counting at least 300 particles, showed that 45.5% of the GNPs were in the 20 to 30 nm range. In addition, it was found that the best density for the used GNPs was 50 μM.

**Figure 1:**
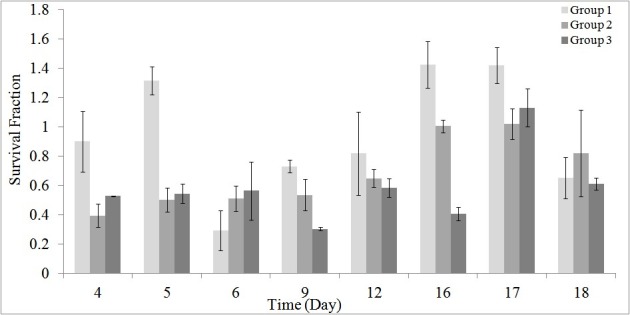
Cell’s survival derived from the MTT assay for the groups 1, 2 and 3. Error bars indicate the standard deviation of the survival fractions of cells in wells for each group.

**Figure 2:**
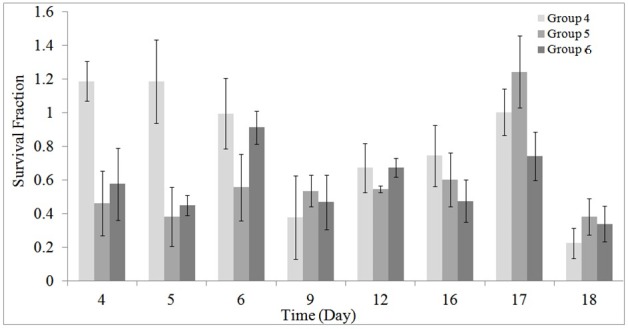
Cell’s survival derived from the MTT assay for the groups 4, 5 and 6.

**Figure 3:**
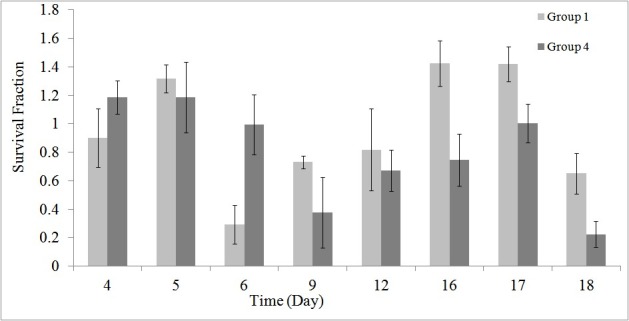
Comparison of the effects of GNPs on cell’s survival.

**Figure 4:**
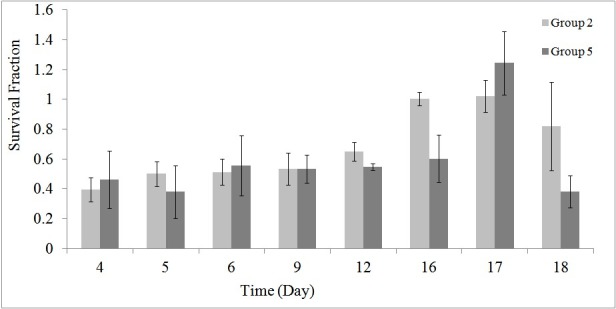
Effects of 1 hour/day irradiation on the cell’s survival in the absence (group 2) and presence (group 5) of GNPs.

**Figure 5:**
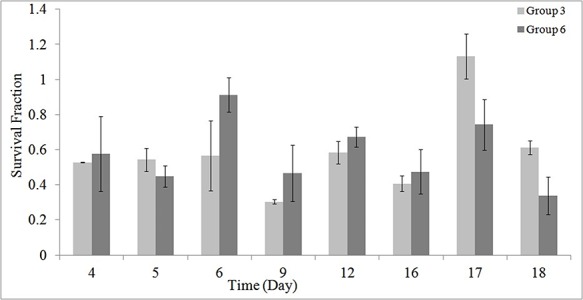
Effects of 2 hour/day irradiation on the cell’s survival in the absence (group 2) and presence (group 5) of GNPs.

As stated earlier, using the MTT assay the survival of the HEK cells in different groups was investigated during and post-irradiation period. The results of MTT assay are shown in Figures 1 to 5.

Figure 1 shows the results of MTT assay on cell’s survival for the studied groups 1, 2 and 3.

Figure 2 illustrates the results of MTT assay on cell’s survival for the studied groups 4, 5 and 6.

Figure 3 gives a comparison between groups 1 and 4 in order to investigate the effects of GNPs on cell death. As stated earlier, for these 2 groups (groups 1 and 4) no radiation was applied to the cells.

Figures 4 and 5 demonstrates the synergic effects of GNP and RF, at 1 and 2 hour/day irradiation, respectively.

Using the ANOVA test, a significant decrease in cell’s survival was seen in the RF exposed group 3 compared to the control group (*P* = 0.035). While, differences were not significant between RF exposed group 2 and the control group (*P* > 0.050). Table 2 shows the mean ± SD of differences of the cell’s survival for groups 1 to 3. In other words, the present study showed that, the cell’s survival in the assay was affected by 8 days, 2 hour/day radiation exposure from 900 MHz RF simulator. Differences were not significant between the 1 and 2 hour/day irradiated groups (*P* > 0.05).

**Table 2:** Comparison of the mean ± SD of differences of the cell’s survival for groups 1 to 3

** Group (I)**	** Group (J) **	**Mean Difference (I-J)**	** SE **	**P**
2	1	-0.1971	0.05896	0.072
3	1	-0.2608	0.05896	0.035

Abbreviation: SE, standard error.

A significant decrease in cell’s survival in the RF exposed groups 5 (*P* = 0.025) and 6 (*P* = 0.018) at the presence of GNP compared to the control group was seen. Table 3 shows the mean ± SD of differences of the cell’s survival for groups 5 to 6 compared to the group 4.

**Table 3:** Comparison of the mean ± SD of differences of the cell’s survival for groups 4 to 6

** Group (I)**	**Group (J)**	**Mean Difference (I-J)**	**SE **	**P**
5	4	-0.1391	0.04564	0.025
6	4	-0.1480	0.04564	0.018

Abbreviation: SE, standard error.

These results indicated that, the cell’s survival in the assay was affected by synergic effects of GNPs and 8 days, 1 to 2 hour/day radiation exposure from 900 MHz RF simulator.

## Discussion

The probability of cure of RCC patients using nephrectomy treatment is mainly related to the stage of cancer (1,3). In addition, due to other affecting factors such as renal insufficiency, solitary kidney, and high-risk comorbidities many patients deemed unsuitable for nephrectomy (1,3). Considering the limitations associated with RCC treatment, in this study, a novel method of cancer treatment is presented using RF radiation in the presence of GNPs.

Results showed that, RF radiation in the presence of GNPs can be used for treatment of RCC with a good efficiency and outcome (Figure 2 and Table 3). The used RF power was 1.0 W at 1 to 2 hour/day radiation and the size and concentrations of GNPs were 20 to 30 and 50 μM, respectively. Furthermore, it was found that, RF radiation induced a significant decrease in cell’s survival when applied for 2 hour/day (Figure 1 and Table 2). Although, differences were not significant for 1 hour/day irradiation, but at the presence of GNPs, a significant decrease in the cell’s survival was seen (*P* = 0.025).

It has been stated that, there is a need to investigate the results of exposure to nanoparticles before any potential therapeutic applications (26). Therefore, in this work, to determine the non-toxic nanoparticles concentration and incubation time the cells were incubated with fresh medium containing different concentrations (0 to 80 μM) of GNPs for 1 to 2 hour. The cells’ survival was investigated after 24 hours post-incubation and then compared with the control group.

Moreover, as stated earlier, GNPs size is another issue that plays an important role on the outcome of treatment (15). In this context, Chen et al studied the toxicity of wide size range of injected GNPs with spheres of diameter 3 to 100 nm in mice (27). They found that, at the dose they were used, the smallest sizes (less than 5 nm) and the largest size (50 to 100 nm) of GNPs are not toxic (27). However, they stated that the intermediate size range of 8 to 37 nm had lethal effects on mice (27). They reported that systematic toxicity of this size range was linked to major organ damage in the liver, spleen, and lungs (27). While, in the same study, the same lethal nanoparticles were not toxic at in vitro condition on Hela cell lines (27). This study demonstrated a large inconsistency between the in vitro and in vivo results, and highlights the concept that simple in vitro experiments may not lead to good predictions regarding in vivo results. However, in this work, due to limitations and difficulties associated with in vivo studies, an in vitro experiment was performed to investigate the effects of RF radiation in the presence of GNPs for the treatment of RCC. Considering that this is the first study dealing with the mentioned possible effects, further in vivo studies on mammalians are of utmost importance. Hence, follow up in vivo studies in larger series, whether on animals or human beings, may provide more convincing evidence.

Recently, RF ablation has become a useful technique for kidney local tumor ablation. This causes the neoplasms destruction by frictional heat applying alternating current (28). Nonetheless, in this study, 900 MHz RF was used for the irradiation of the cell. RF at such radiation can affect the kidney tumor by a specific non-thermal action (29). Few studies have demonstrated the non-thermal levels of RF radiation on brain (29). They have measured the temperature before and after but not during exposure to RF (29). With respect to such non-thermal levels of RF effects, Panagopoulos et al studied the mechanism of RF action on cells plasma membrane using a biophysical model (30). Their results showed that, an external EMF causes forced-vibration of all the free ions on the membrane surface (30).

## Conclusion

In this study the effect of RF radiation in the presence of GNPs, as a novel treatment method for RCC treatment was evaluated. Results showed that, this method can be efficiently used for RCC treatment as an alternative of nephrectomy. More follow up in vivo studies on mammalians are needed to investigate the potential of the presented method for clinical applications.

## Limitations of the study

In this work, few treatment regimens based on synergetic effects of GNPs and RF radiation were followed. The results here should be confirmed in larger series, employing repeated GNPs incubation size and time related effects and providing different RF exposure dose rates.

## Acknowledgments

The authors would like to thank Dr. Shiva Moein from Genetic Department, Isfahan University of Medical Sciences, Isfahan, Iran for her critical comments and technical advices.

## Authors’ contribution

SN, GM and MBGh were the principal investigators of the study. AH, SN, MM, YGh, SMS and MBGh participated in preparing the concept and design. LHN participated in nano-particle synthesis. SN, GM and MBGh revisited the manuscript and critically evaluated the intellectual contents. All authors participated in preparing the final draft of the manuscript, revisited the manuscript and critically evaluated the intellectual contents. All authors have read and approved the content of the manuscript and confirmed the accuracy or integrity of any part of the work.

## Conflicts of Interest

The authors report no conflicts of interest. The authors alone are responsible for the content and writing of this paper.

## Ethical considerations

Ethical issues (including plagiarism, data fabrication, double publication) have been completely observed by the authors.

## Funding/Support

The study was supported by a grant from Sabzevar University of Medical Sciences (grant number #1394-157).
